# Editorial: Promoting innovative learning-oriented feedback practice: engagement, challenges, and solutions

**DOI:** 10.3389/fpsyg.2025.1707902

**Published:** 2025-10-06

**Authors:** Wei Wei

**Affiliations:** Faculty of Applied Sciences, Macao Polytechnic University, Macao, China

**Keywords:** innovative feedback, feedback engagement, self feedback, GenAI feedback, learning oriented assessment

## Introduction

The studies on feedback have experienced some fundamental changes: from identifying features of effective feedback to exploring how it is delivered and constructed, from focusing on feedback givers (teachers, peers, computers, and, more recently, GenAI tools) to its receivers or users, and from portraying feedback as a one-way action from givers to receivers to a more collaborative process between them that emphasizes learners' feedback-seeking initiatives. During these significant shifts, students' engagement patterns with feedback have been at the center of discussions. Engagement patterns in technology-assisted feedback refer to how learners interact with and respond to feedback provided through digital tools, encompassing their cognitive processes, emotional responses, and behaviors when receiving and applying feedback in educational settings.

Without exception, the articles in this special topic present an excellent summary of these shifts from multiple perspectives. This Research Topic assembles seven scholarly articles from diverse international contexts, including Mainland China, Vietnam, Norway, and Sweden. These studies explore and examine different aspects of innovative feedback practice across a wide range of disciplines, such as mathematics, languages, engineering, and medicine. Moving beyond the traditional view of feedback as the transmission of information from one side to the other to narrow the gap between current performance and expected outcomes, these articles have highlighted key themes in designing and promoting innovative feedback practice, which can be broadly categorized into three closely interlinked parts: learners' characteristics, interactions and engagement with feedback, and scaffolding and learning environments.

## The characteristics of learners

A frequently quoted framework in the design and analysis of feedback studies is the student-feedback interaction model (Lipnevich and Smith, 2022). This model lists six learner characteristics as contributing factors to students' further intention or engagement level with received feedback: (1) ability, (2) receptivity, (3) expectations, (4) self-efficacy, (5) motivation, and (6) personality. Specifically, these individual differences influence a learner's engagement with and response to feedback, thereby shaping the effectiveness of the feedback process. In this special Research Topic, Söderström et al. found that mastery goals (which focus on the development of competency and skills) have a direct positive influence on students' perceived usefulness of received feedback, while performance goals (which are related to a motivation to stand out from peers competitively) do not show similar effects. Furthermore, rather than highlighting the direct impact of self-efficacy on feedback engagement, Zhang et al.'s study of self-efficacy reported a mediation model. Their research found that self-efficacy mediates the influence of pressure—specifically, challenge time pressure and hindrance time pressure—on students' self-reported level of innovative behaviors in postgraduate studies, including the communication and engagement with feedback from both peers and supervisors.

## The interactions and engagement

As for the second emerging theme in this special Research Topic of studies, research on learners' interaction and engagement patterns with feedback now centers on students' cognitive, affective, and behavioral processing of it, and the interplay among these processes. In this Research Topic, Yang et al. went beyond the traditional view of engaging with feedback from outsiders (i.e., peers, teachers, and learning analytics) and attempted to validate a research instrument to measure learners' self-feedback behaviors. The three confirmed dimensions (seeking, processing, and using feedback) can greatly benefit researchers and teachers seeking to better understand and design pedagogical activities that scaffold self-feedback practice. Moreover, in the context of self-regulated online learning, Dao et al. placed more emphasis on the role of cognitive engagement. Their study involved designing an online laboratory system in which learners can analyze and set up learning goals, discuss them with peers, and resubmit their work. These embedded activities and mechanisms within the laboratory system not only serve as a reminder but also, more importantly, as scaffolding to help Asian learners overcome their emotional and cognitive barriers to engaging with formative peer feedback before they final submission of their work.

## Scaffolding and learning environments

Last but not least, previous frameworks have not elaborated on the role of technology in providing sufficient scaffolding for feedback. Recently, generative AI has been identified as having the capability to offer such feedback, including instructions for learning, developing learning plans, and recommending learning strategies (Chen et al., 2025a). However, this capability requires a supportive learning environment to be effective. As noted by Chen et al. (2025b), when AI-generated feedback does not align with the expectations or preferences of peers and teachers, students are less likely to accept it. In this Research Topic, three studies offered more details on how a facilitating environment may promote effective feedback practice. For instance, Zhang et al.'s study on PhD students revealed that supervisors' support leads to greater innovative learning behaviors at the postgraduate level, such as discussing more frequently and actively seeking feedback from both peers and supervisors. Moreover, although learning routines and established teaching practices can hardly be challenged, Chen et al. attempted to introduce a new teaching pedagogy to combat their negative consequences. Learners were given some degree of autonomy in discussing, learning from others' contributions, and summarizing their learning progress independently. Finally, Lu et al. studied collaborative problem-solving skills (CPS) in the mathematics classroom and argued that identifying, diagnosing, and visualizing cognitive conflicts during the CPS process can help teachers adopt a learning-oriented approach (rather than an evaluative one) and create opportunities for learners to provide timely peer feedback.

As for future studies, the arrival of GenAI has revolutionized the feedback generation and feedback engagement process on a large scale. However, researchers have issued an increasing number of concerns, as working with GenAI requires effective prompting, which presents another challenge to learners. On the bright side, those who understand expected outcomes, have a higher level of AI literacy, and have the mindset to explore and evaluate GenAI-generated feedback may have a greater advantage in benefiting from GenAI-human collaborations. However, metacognitive laziness and overreliance, which result from blindly accepting GenAI feedback without conducting task analysis and monitoring the alignment between AI output and task demands, have already started to erode learners' academic achievements (Fu et al., 2025). Strategies for developing AI literacy and solutions for combating these undesired consequences of GenAI are urgently needed by both learners and educators.
